# Short- versus long-segment posterior spinal fusion with vertebroplasty for osteoporotic vertebral collapse with neurological impairment in thoracolumbar spine: a multicenter study

**DOI:** 10.1186/s12891-020-03539-0

**Published:** 2020-08-01

**Authors:** Yuya Ishikawa, Kei Watanabe, Keiichi Katsumi, Masayuki Ohashi, Yohei Shibuya, Tomohiro Izumi, Toru Hirano, Naoto Endo, Takashi Kaito, Tomoya Yamashita, Hiroyasu Fujiwara, Yukitaka Nagamoto, Yuji Matsuoka, Hidekazu Suzuki, Hirosuke Nishimura, Hidetomi Terai, Koji Tamai, Atsushi Tagami, Shuta Yamada, Shinji Adachi, Toshitaka Yoshii, Shuta Ushio, Katsumi Harimaya, Kenichi Kawaguchi, Nobuhiko Yokoyama, Hidekazu Oishi, Toshiro Doi, Atsushi Kimura, Hirokazu Inoue, Gen Inoue, Masayuki Miyagi, Wataru Saito, Atsushi Nakano, Daisuke Sakai, Tadashi Nukaga, Shota Ikegami, Masayuki Shimizu, Toshimasa Futatsugi, Seiji Ohtori, Takeo Furuya, Sumihisa Orita, Shiro Imagama, Kei Ando, Kazuyoshi Kobayashi, Katsuhito Kiyasu, Hideki Murakami, Katsuhito Yoshioka, Shoji Seki, Michio Hongo, Kenichiro Kakutani, Takashi Yurube, Yasuchika Aoki, Masashi Oshima, Masahiko Takahata, Akira Iwata, Hirooki Endo, Tetsuya Abe, Toshinori Tsukanishi, Kazuyoshi Nakanishi, Kota Watanabe, Tomohiro Hikata, Satoshi Suzuki, Norihiro Isogai, Eijiro Okada, Haruki Funao, Seiji Ueda, Yuta Shiono, Kenya Nojiri, Naobumi Hosogane, Ken Ishii

**Affiliations:** 1 Department of Orthopaedic Surgery, Niigata University, 1-757 Asahimachidori, Chuo-ku, Niigata City, Niigata, 951-8510 Japan; 2 Department of Orthopaedic Surgery, Osaka University, 2-2 Yamadaoka, Suita City, Osaka 565-0871 Japan; 3 Department of Orthopaedic Surgery, Tokyo Medical University, 6-1-1 Shinjuku, Shinjuku-ku, Tokyo, 160-8402 Japan; 4 Department of Orthopaedic Surgery, Osaka City University, 1-4-3 Asahimachi, Abeno-ku, Osaka, 545-8585 Japan; 5 Department of Orthopaedic Surgery, Nagasaki University, 1-7-1 Sakamoto, Nagasaki City, Nagasaki, 852-8501 Japan; 6 Department of Orthopaedic Surgery, Tokyo Medical and Dental University, 1-5-45 Yushima, Bunkyo-ku, Tokyo, 113-8519 Japan; 7 Department of Orthopaedic Surgery, Kyushu University, 3-1-1 Maidashi, Higashi-ku, Fukuoka City, 812-8582 Japan; 8 Department of Orthopaedic Surgery, Jichi Medical University, 3311-1 Yakushiji, Shimotsuke City, Tochigi 329-0498 Japan; 9 Department of Orthopaedic Surgery, Kitasato University, 1-15-1 Kitasato, Minami-ku, Sagamihara City, Kanagawa 252-0374 Japan; 10 Department of Orthopaedic Surgery, Osaka Medical College, 2-7 Daigakumachi, Takatsuki City, Osaka 569-0801 Japan; 11 Department of Orthopaedic Surgery, Tokai University, 143 Shimokasuya, Isehara City, Kanagawa 259-1193 Japan; 12 Department of Orthopaedic Surgery, Shinshu University, 3-1-1, Asahi, Matsumoto City, Nagano 390-8621 Japan; 13 Department of Orthopaedic Surgery, Chiba University, 1-8-1 Inohana, Chuo-ku, Chiba City, 260-8670 Japan; 14 Department of Orthopaedic Surgery, Nagoya University, 65 Tsurumai-cho, Showa-ku, Nagoya City, Aichi 466-8560 Japan; 15 Department of Orthopaedic Surgery, Kochi University, Oko-cho Kohasu, Nankoku City, Kochi 783-8505 Japan; 16 Department of Orthopaedic Surgery, Nagoya City University, 1 Kawasumi, Mizuho-cho, Mizuho-ku, Nagoya, 467-8601 Japan; 17 Department of Orthopaedic Surgery, Kanazawa University, 13-1 Takaramachi, Kanazawa City, Ishikawa 920-8641 Japan; 18 Department of Orthopaedic Surgery, University of Toyama, 2630 Sugitani, Toyama City, Toyama, 930-0194 Japan; 19 Department of Orthopaedic Surgery, Akita University, 1-1-1 Hondo, Akita City, 010-8543 Japan; 20 Department of Orthopaedic Surgery, Kobe University, 7-5-1 Kusunoki-cho, chuou-ku, Kobe City, Hyogo 650-0017 Japan; 21 Department of Orthopaedic Surgery, Eastern Chiba Medical Center, 3-6-2 Okayamadai, Togane City, Chiba 283-8686 Japan; 22 Department of Orthopaedic Surgery, Nihon University Itabashi Hospital, 30-1 Oyaguchikamicho, Itabashi-ku, Tokyo, 173-8610 Japan; 23 Department of Orthopaedic Surgery, Hokkaido University, North-15, West-7, Kita-ku, Sapporo City, Hokkaido 060-8638 Japan; 24 Department of Orthopaedic Surgery, Iwate Medical University, 19-1 Uchimaru, Morioka City, Iwate 020-8505 Japan; 25 Department of Orthopaedic Surgery, University of Tsukuba, 1-1-1 Tennodai, Tsukuba City, Ibaraki 305-8577 Japan; 26 Department of Orthopaedic Surgery, Hiroshima University, 1-2-3 Kasumi, Minami-ku, Hiroshima City, Hiroshima, 734-8551 Japan; 27 Department of Orthopaedic Surgery, Keio University School of Medicine, 35 Shinanomachi, Shinjuku-ku, Tokyo, 160-8582 Japan; 28 Department of Orthopaedic Surgery, International University of Health and Welfare School of Medicine, Mita, Minato-ku, Tokyo, 108-8329 Japan; 29 Department of Orthopaedic Surgery, National Defense Medical College, 3-2 Namiki, Tokorozawa City, Saitama, 359-8513 Japan; 30 Department of Orthopaedic Surgery, School of Medicine, International University of Health and Welfare, Mita, Minato-ku, Tokyo, 108-8329 Japan

**Keywords:** Osteoporotic vertebral collapse, Vertebral fracture, Thoracolumbar spine, Vertebroplasty, Posterior spinal fusion, Short-segment, Long-segment, Correction loss, Kyphosis

## Abstract

**Background:**

Vertebroplasty with posterior spinal fusion (VP + PSF) is one of the most widely accepted surgical techniques for treating osteoporotic vertebral collapse (OVC). Nevertheless, the effect of the extent of fusion on surgical outcomes remains to be established. This study aimed to evaluate the surgical outcomes of short- versus long-segment VP + PSF for OVC with neurological impairment in thoracolumbar spine.

**Methods:**

We retrospectively collected data from 133 patients (median age, 77 years; 42 men and 91 women) from 27 university hospitals and their affiliated hospitals. We divided patients into two groups: a short-segment fusion group (S group) with 2- or 3-segment fusion (87 patients) and a long-segment fusion group (L group) with 4- through 6-segment fusion (46 patients). Surgical invasion, clinical outcomes, local kyphosis angle (LKA), and complications were evaluated.

**Results:**

No significant differences between the two groups were observed in terms of neurological recovery, pain scale scores, and complications. Surgical time was shorter and blood loss was less in the S group, whereas LKA at the final follow-up and correction loss were superior in the L group.

**Conclusion:**

Although less invasiveness and validity of pain and neurological relief are secured by short-segment VP + PSF, surgeons should be cautious regarding correction loss.

## Background

The number of patients with osteoporotic vertebral fractures is continuously increasing with the aging of society [1–3]. Most fractures are expected to heal conservatively; however, in some cases, fractured vertebrae lead to insufficient union and acquire intravertebral instability. As a result, vertebral bodies progress to delayed collapse, a process referred to as osteoporotic vertebral collapse (OVC), causing protrusion of bony fragments into spinal canal and segmental kyphosis, and ultimately causing neurological deficits [4]. In these cases, surgical procedures are recommended, including anterior reconstruction [5], posterior spinal fusion [6], anterior and posterior combined surgery [7], posterior spinal shortening [8, 9], and vertebroplasty with posterior spinal fusion (VP + PSF) [10–16].

VP + PSF is one of the most widely accepted procedures that achieves neurological recovery and pain relief; compared to other procedures, VP + PSF is less invasive but tends to be associated with greater correction loss [7, 17–19]. Nevertheless, because of a lack of evidence regarding the extent of VP + PSF for OVC with neurological impairment, no consensus has been reached regarding how many spinal segments should be fused using this procedure. Therefore, in this study, we investigated the effect of fusion extent on surgical outcomes of VP + PSF for treating OVC with neurological impairment in the thoracolumbar spine.

## Methods

This study was reviewed and approved by the institutional review board of all institutions involved. This was a retrospective study of patients with OVC with neurological impairment who underwent surgical intervention from 2005 to 2014 at 27 university hospitals and their affiliated hospitals, which participated in the Japan Association of Spine Surgeon with Ambition multicenter database. Of the 406 patients who underwent surgery for OVC, patients who met the following criteria were included: 1) affected thoracolumbar junction, from T10 to L2; 2) existence of neurological impairments, including motor weakness or neuralgia in the lower extremity; 3) vertebroplasty with instrumented posterior spinal fusion; and 4) minimum 2-year follow-up after surgery. Patients undergoing surgery for several vertebral fractures, and patients suffered from the fracture by high-energy trauma were excluded. In total, 133 patients, including 42 men and 91 women, were included. The patients were divided into two groups depending on the number of fused segments. The short-segment fusion group (S group) included 87 patients who underwent 2- or 3-segment fusion. The long-segment fusion group (L group) included 46 patients who underwent 4-, 5-, or 6-segment fusion.

### Surgical procedure

The surgical procedure consists of posterior fixation, which used pedicle screws and rod system, and vertebroplasty, which filled hydroxyapatite blocks or bone cement into vertebrae via the transpedicular approach. The extent of posterior fusion area was decided depending on institutional or surgeon’s experience. In some cases, neural decompression was performed using laminectomy. Depending on the institutional policy or surgeons’ decision, laminar hooks or sublaminar wiring were used under some circumstances. All patients underwent open surgery with grafting autografts and/or artificial bones posteriorly.

### Clinical evaluation

We reviewed patient characteristics, surgical invasion, clinical outcomes, and complications from medical charts in each institution and recorded the data in a predetermined common format. We evaluated preoperative status and clinical results at the point of final medical examination. Patient characteristics included age, sex, affected vertebra, the number of comorbidities, secondary osteoporosis, and preoperative medication with an osteoporosis drug. Comorbidities included cardiac disease, pulmonary disease, renal failure, hepatitis, collagen disease, cancer, Parkinson disease, mental disorders, stroke, neuromuscular disease, and diabetes mellitus. We defined secondary osteoporosis as the prevalence of diabetes mellitus, rheumatoid arthritis, chronic renal failure, post-gastrectomy malabsorption, or oral intake of steroids, anticonvulsants, warfarin, or selective serotonin reuptake inhibitors. We considered the patients who did not present with the above characteristics as having primary osteoporosis. The evaluation of surgical invasion included surgical time and intraoperative blood loss.

Clinical outcomes were evaluated using the Japanese Orthopaedic Association scoring system ([JOA score], ranging from 0 [worst condition] to 15 [best condition]) [20]; walking ability was rated using the following scoring system: score 1, independent walking; score 2, dependent walking with a cane; score 3, dependent walking with walker; and score 4, unable to walk. The visual analog scale (VAS) was used for low back pain and lower extremity pain or numbness (ranging from 0 [no symptoms] to 100 [worst symptoms]). The recovery rate of the JOA score was calculated using Hirabayashi’s method as follows: ([final follow-up score–preoperative score]/[15–final follow-up score] × 100).

### Radiological evaluation

**Fig. 1:**
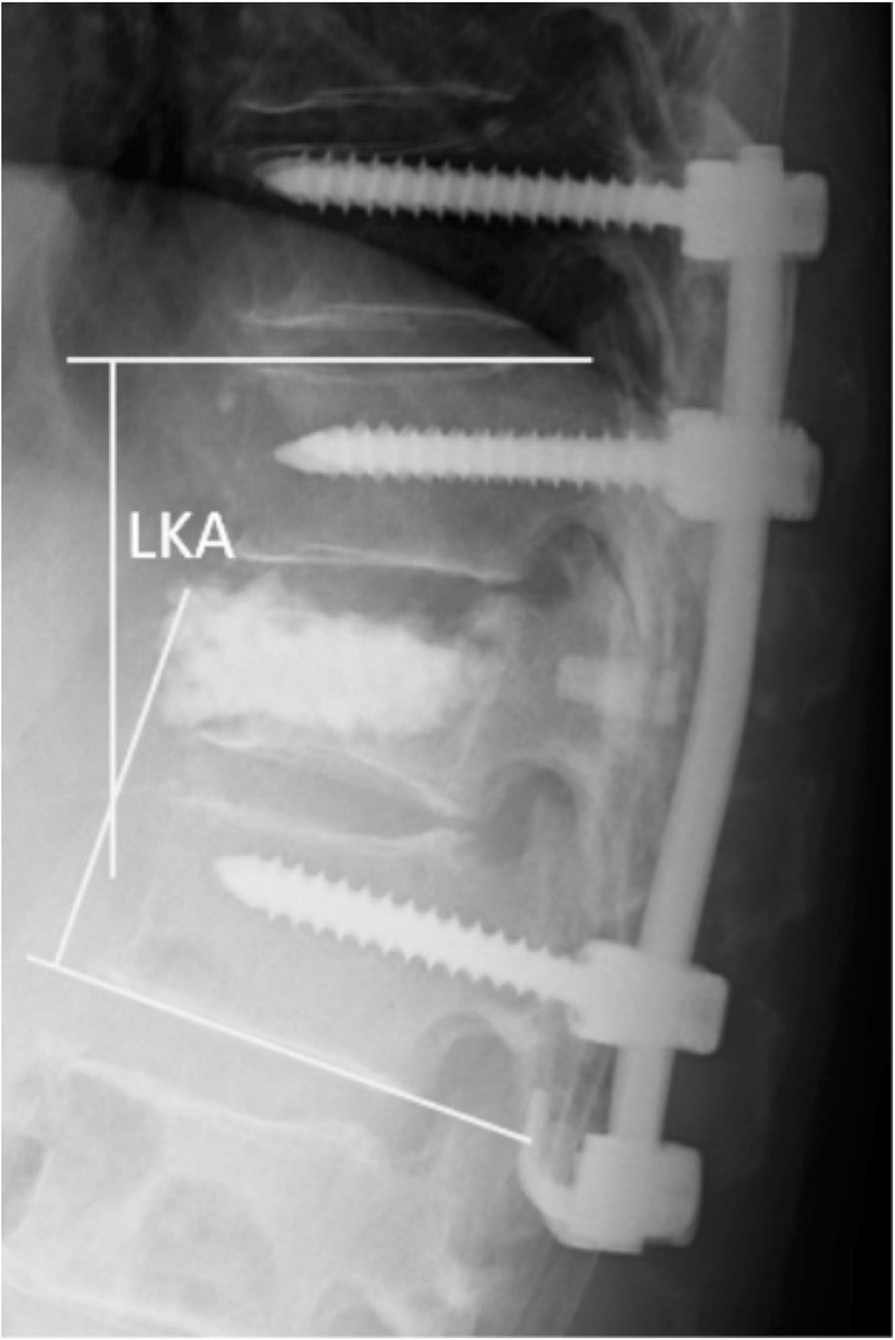
Using lateral radiographs, local kyphosis angle (LKA) was measured between the upper endplate of the vertebra one above the affected level and the lower endplate of the vertebra one below the affected level using the Cobb method

Plain radiographs were taken preoperatively, postoperatively, and at the point of final examination in all patients. Typically, radiographs were taken in a standing posture. However, in patients who were unable to ambulate, a lateral decubitus or sitting posture were accepted in replacement. On the lateral view of plain radiographs, we measured local kyphosis angle (LKA) between the upper endplate of the vertebra, one above the affected level, and the lower endplate of the vertebra, one below the affected level using the Cobb method (Fig. 1). We calculated correction angle as follows: (postoperative LKA–preoperative LKA) and correction loss angle: (LKA at final follow-up–postoperative LKA).

### Complications

We reviewed perioperative complications, such as neurological deterioration, dural tears, hematomas, delirium, pneumonia, cardiac disease, surgical site infections, gastrointestinal disease, venous thrombosis, and electrolyte abnormalities that occurred within 6 weeks after surgery. Requirements for reoperation and the details of these procedures were also assessed.

In addition, we evaluated the presence of pedicle screw back-out, fracture of the uppermost or lowermost instrumented vertebra, and vertebral fracture adjacent to uppermost or lowermost instrumented vertebra on plain radiographs.

### Statistical analysis

Categorical and continuous variables were analyzed using Fisher’s exact test and Mann–Whitney U test, respectively, using GraphPad Prism7 (GraphPad Software). A *p* value < 0.05 was considered statistically significant. Values were presented as median [interquartile range (IQR): 25–75%].

## Results

### Patient demographics

Demographic data of the patients are shown in Table 1. There were no statistically significant differences between the two groups.

**Table 1:** Comparison of patients demographics

Variables	S group	L group	*p* value
Number of the patients	87	46	
Detail of fused segments: segments (patients) (n)	2 (26) 3 (61)	4 (27) 5 (11) 6 (8)	
Age at surgery	77 [72–80]	77 [73–82]	0.42
Men/women (n)	28/59	14/32	1
Affected vertebra (n)			0.59
Th10	3	3	
Th11	7	5	
Th12	36	18	
L1	31	18	
L2	10	2	
Number of comorbidities	1 [0–1]	1 [0–2]	0.55
Secondary osteoporosis (%)	40.2	34.8	0.58
Preoperative medication of the osteoporosis drug (%)	28.7	41.3	0.18
Preoperative steroid administration (%)	6.9	10.9	0.51
F/u period (mon)	43 [30–59]	37 [30–54]	0.56

Continuous variables are shown as median [IQR: 25–75%]

*Abbreviations*: *S*, short-segment fusion; *L*, long-segment fusion; *F/u*, follow up

### Surgical invasion

**Fig. 2:**
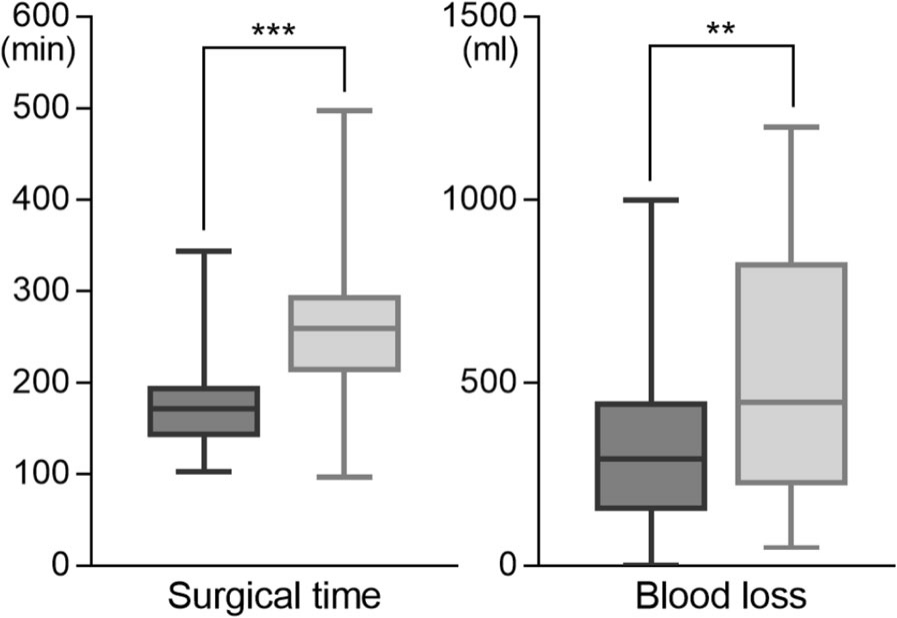
Surgical time and blood loss in the S and L groups. Each box indicates interquartile range and line in the box indicates median value. Bar is minimum to maximum; ** *p* < 0.01, *** *p* < 0.001

The surgical times were 172 [IQR: 141–195] min and 260 [IQR: 213–292] min in the S and L groups, respectively; the intraoperative blood loss was 293 [IQR: 150–450] ml and 448 [IQR: 220–830] ml in the S and L groups, respectively (Fig. 2). In the S group, the surgical time was significantly shorter (*p* < 0.001) and the intraoperative blood loss was significantly less (*p* = 0.001).

### Clinical evaluation

Clinical outcomes comparing the two groups are shown in Table 2. Although preoperative JOA score was significantly worse in the L group than in the S group (*p* = 0.009), the score at final follow-up and the recovery rate were not significantly different. Regarding walking ability grade, there were no significant differences preoperatively and at the final follow-up between the two groups. Scores for low back pain and low extremity pain also showed no significant differences.

**Table 2:** Comparison of outcomes

Variables	S group	L group	*p* value
LBP score (0–100)			
Pre	80 [65–90]	80 [60–90]	0.62
Final	30 [10–50]	28 [10–50]	0.88
LEP score (0–100)			
Pre	50 [10–70]	50 [15–80]	0.54
Final	10 [0–30]	10 [0–30]	0.10
JOA score (0–15)			
Pre	6 [3–7]	4 [1–6]	< 0.01**
Final	10 [8–12]	10 [6–12]	0.67
Recovery rate	50.0 [28.6–67.3]	50.0 [24.5–73.3]	0.62
Walking ability (1–4)			
Pre (%)			
1	8.1	2.2	0.41
2	11.5	4.3	
3	24.1	34.8	
4	56.3	58.7	
Final (%)			
1	40.2	34.8	0.35
2	31.0	23.9	
3	19.5	34.8	
4	9.2	6.5	
Correction angle (°)	12 [7–19]	16 [7–23]	0.14
Correction loss (°)	9 [4–13]	4 [0–10]	< 0.01**

Continuous variables are shown as median [IQR: 25–75%]

** *p* < 0.01

*Abbreviations*: *LBP,* low back pain; *LEP,* lower extremity pain; *JOA*, Japanese Orthopaedic Association

### Radiological evaluation

**Fig. 3:**
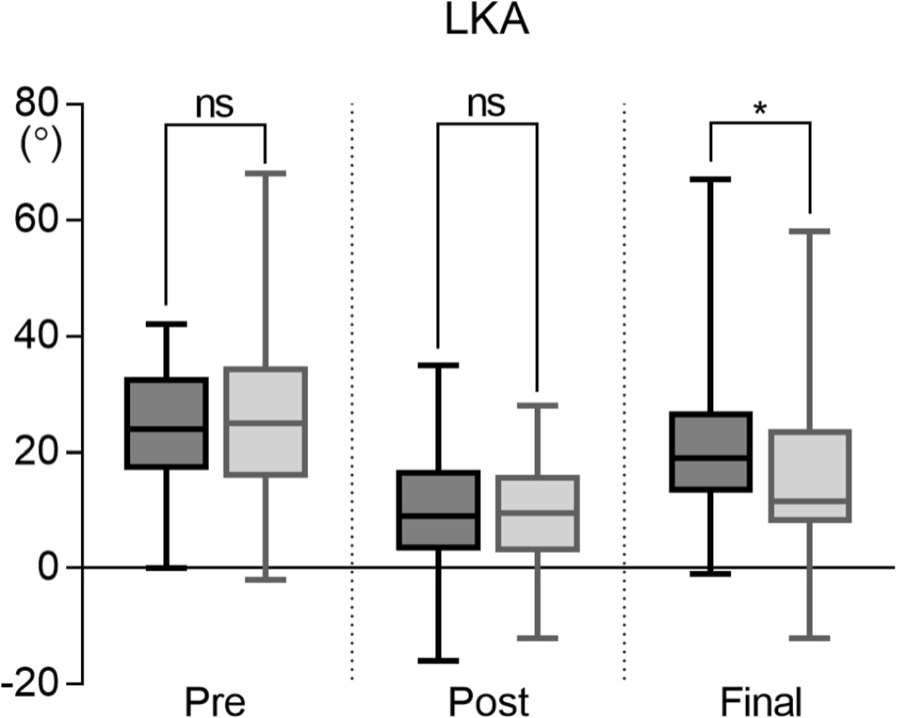
Changes in local kyphosis angle preoperatively, postoperatively, and at final follow-up in the S and L groups; *ns* not significant, * *p* < 0.05

In the S group, LKA was 24° [IQR: 18–33°] preoperatively, 9° [IQR: 4–17°] immediately after surgery, and 19° [IQR: 14–27°] at the final follow-up; in the L group, LKA was 25° [IQR: 16–34°] preoperatively, 10° [IQR: 3–16°] postoperatively, and 12° [IQR: 8–24°] at final follow-up (Fig. 3). Although preoperative and postoperative LKA were comparable between the two groups, LKA at final follow-up was significantly smaller in the L group than in the S group (*p* = 0.017). Correction angle and correction loss are shown in Table 2. Correction angle showed no significant difference between the groups, whereas correction loss was greater in the S group than in the L group. The maximum value of correction loss was 54° in the S group and 36° in the L group.

### Complications

Complications observed in each group are shown in Table 3. No significant difference was found between the groups in terms of incidence of perioperative complications, mechanical complications, or reoperation rates. The details of the reoperations are shown in Table 3.

**Table 3:** Comparison of complications

Variables	S group	L group	*p* value
Perioperative complications (%)	16.1	15.2	1
Neurological deterioration (%)	0	0	
Dural tear (%)	1.1	2.2	1
Hematoma (%)	0	2.2	0.35
Delirium (%)	8.0	0	0.095
Pneumonia (%)	1.1	0	1
Cardiac disease (%)	0	2.2	0.35
SSI (%)	2.3	4.3	0.61
Gastrointestinal disease (%)	1.1	4.3	0.28
Venous thrombosis (%)	1.1	0	1
Electrolyte abnormality (%)	1.1	0	1
Fracture of UIV or LIV (%)	20.7	15.2	0.49
VF adjacent to UIV or LIV (%)	16.3	19.6	0.64
Screw cut out (%)	6.9	10.9	0.51
Reoperation (%)	11.5	10.9	1
Implant removal due to screw cut out (%)	1.1	4.3	
Extension of PSF (%)	6.9	4.3	
Anterior reconstruction and extension of PSF (%)	3.4	2.2	

*Abbreviations*: *SSI* surgical site infection, *UIV* uppermost instrumented vertebra, *LIV* lowermost instrumented vertebra, *VF* vertebral fracture, *PSF* posterior spinal fusion

## Discussion

In this retrospective multicenter study, we evaluated surgical results of short- versus long-segment VP + PSF for treating OVC with neurological impairment in the thoracolumbar spine. Neurological recovery, pain scale, and complications were almost the same with both procedures. Surgical time was shorter and blood loss was less in the S group, whereas LKA at the final follow-up and correction loss were superior in the L group. Our findings underscore the effect of fusion extent on surgical outcomes of VP + PSF for treating OVC.

The advantages of short-segment fusion are its lower degree of invasiveness that would be more favorable for elderly patients and preservation of more motion segments. In contrast, short-segment fusion generates concerns about provoking excessive load to the constructs, leading to early implant failure and loss of sagittal alignment [21]. Regarding rigidity after posterior spinal instrumentation, the longer the spinal segments are instrumented, the more stability appears to be acquired. In biomechanical studies on thoracic [22] and thoracolumbar vertebral fracture models [23] that are stabilized with pedicle screws and rods, two-levels above and below constructs provided more stability than one-level above and below constructs.

In clinical studies comparing short- versus long-segment fixation, limited evidence is available regarding thoracolumbar burst fractures. Tezeren and Kuru [24] and Altay et al. [25] showed similar clinical outcomes of short- and long-segment fixation, although sagittal radiographic parameters were superior for long-segment fixation. Ugras et al. [26] reported that procedures preserving one lumbar segment showed no differences from those of long-segment fixation with respect to both clinical and radiographic outcomes.

Regarding OVC, evidence on the effect of the extent of fusion segments on surgical outcomes has been poorly documented. Because these osteoporotic patients have fragile bones, there have been some apprehension as to whether short-segment fusion would provide sufficient stability. In our study, JOA score recovery, pain relief, walking ability, complications in the perioperative period, and mechanical complications including instrumented vertebral fractures and screw cut-out, showed no difference between the groups, suggesting that short-segment fusion offers almost the same clinical outcomes as long-segment fusion. Meanwhile, in accordance with biomechanical studies, our study suggested that long-segment fusion is superior for the management of LKA. In this regard, if the purpose of surgery is not only pain or neurological recovery but also correcting alignment to appropriate degree, long-segment fusion would be more suitable. Nevertheless, there remains uncertainty regarding the correction of kyphosis, despite excessive invasion because there is no consensus regarding the amount of acceptable residual deformity [27], especially in elderly patients.

Despite of the extent of fusion segment, some patients experienced a large amount of correction loss. To circumvent this, an anterior reconstruction can be added to posterior fixation. However, the surgical invasion would be larger in this case [7, 17–19]. Another potential prevention might be the administration of teriparatide [28, 29], the use of expandable screws [30] or cement augmentation of pedicle screws [31, 32].

Our study has several limitations. There is some institutional bias in surgical indications, procedure, and determination of the extent of fusion segments. Nevertheless, no significant differences were observed between the two groups with respect to age, gender, affected vertebra, secondary osteoporosis, preoperative use of osteoporosis medication, and follow-up period. In this context, it is reasonable to compare these two surgical methods for a retrospective study.

One of the reasons the decision of the surgical technique used varies may be due to the fact that no classification is available for this disorder. To compare the outcomes of the procedures more precisely, there is a great need for a classification for OVC warranting the decision of surgical procedure.

Another limitation is that we evaluated LKA as a sagittal parameter, but not global alignment parameters. Because the concept of global alignment was not sufficiently recognized in early part of the study period, whole spine radiographs were not obtained from some patients. Further investigation is required to determine the acceptable posttraumatic deformities.

## Conclusion

Despite the fact that short-segment VP + PSF showed less invasiveness and validity of pain and neurological relief, more correction loss was observed than for long-segment VP + PSF. Surgeons should be cautious regarding correction loss when performing short-segment VP + PSF.

## Data Availability

The data that support the findings of this study are included within the article and tables. The dataset used and analyzed during this study are available from the corresponding author on reasonable request.

## Abbreviations

VP + PSF: Vertebroplasty with posterior spinal fusion
OVC: Osteoporotic vertebral collapse
LKA: Local kyphosis angle
JOA: Japanese Orthopaedic Association
VAS: Visual analog scale
IQR: Interquartile range

## References

[1] Oinuma T, Sakuma M, Endo N (2010). Secular change of the incidence of four fracture types associated with senile osteoporosis in Sado, Japan: the results of a 3-year survey. J Bone Miner Metab.

[2] Hernlund E, Svedbom A, Ivergard M, Compston J, Cooper C, Stenmark J (2013). Osteoporosis in the European Union: medical management, epidemiology and economic burden. A report prepared in collaboration with the International Osteoporosis Foundation (IOF) and the European Federation of Pharmaceutical Industry Associations (EFPIA). Arch Osteoporos.

[3] Park SB, Kim J, Jeong JH, Lee JK, Chin DK, Chung CK (2016). Prevalence and incidence of osteoporosis and osteoporotic vertebral fracture in Korea: Nationwide epidemiological study focusing on differences in socioeconomic status. Spine (Phila Pa 1976).

[4] Ito Y, Hasegawa Y, Toda K, Nakahara S (2002). Pathogenesis and diagnosis of delayed vertebral collapse resulting from osteoporotic spinal fracture. Spine J.

[5] Kanayama M, Ishida T, Hashimoto T, Shigenobu K, Togawa D, Oha F (2010). Role of major spine surgery using Kaneda anterior instrumentation for osteoporotic vertebral collapse. J Spinal Disord Tech.

[6] Ataka H, Tanno T, Yamazaki M (2009). Posterior instrumented fusion without neural decompression for incomplete neurological deficits following vertebral collapse in the osteoporotic thoracolumbar spine. Eur Spine J.

[7] Nakashima H, Imagama S, Yukawa Y, Kanemura T, Kamiya M, Deguchi M (2015). Comparative study of 2 surgical procedures for osteoporotic delayed vertebral collapse: anterior and posterior combined surgery versus posterior spinal fusion with vertebroplasty. Spine (Phila Pa 1976).

[8] Saita K, Hoshino Y, Kikkawa I, Nakamura H (2000). Posterior spinal shortening for paraplegia after vertebral collapse caused by osteoporosis. Spine (Phila Pa 1976).

[9] Suzuki T, Abe E, Miyakoshi N, Murai H, Kobayashi T, Abe T (2013). Posterior-approach vertebral replacement with rectangular parallelepiped cages (PAVREC) for the treatment of osteoporotic vertebral collapse with neurological deficits. J Spinal Disord Tech.

[10] Matsuyama Y, Goto M, Yoshihara H, Tsuji T, Sakai Y, Nakamura H (2004). Vertebral reconstruction with biodegradable calcium phosphate cement in the treatment of osteoporotic vertebral compression fracture using instrumentation. J Spinal Disord Tech.

[11] Sudo H, Ito M, Abumi K, Kotani Y, Takahata M, Hojo Y (2010). One-stage posterior instrumentation surgery for the treatment of osteoporotic vertebral collapse with neurological deficits. Eur Spine J.

[12] Uchida K, Nakajima H, Yayama T, Miyazaki T, Hirai T, Kobayashi S (2010). Vertebroplasty-augmented short-segment posterior fixation of osteoporotic vertebral collapse with neurological deficit in the thoracolumbar spine: comparisons with posterior surgery without vertebroplasty and anterior surgery. J Neurosurg Spine.

[13] Lee SH, Kim ES, Eoh W (2011). Cement augmented anterior reconstruction with short posterior instrumentation: a less invasive surgical option for Kummell's disease with cord compression. J Clin Neurosci.

[14] Patil S, Rawall S, Singh D, Mohan K, Nagad P, Shial B (2013). Surgical patterns in osteoporotic vertebral compression fractures. Eur Spine J.

[15] Katsumi K, Hirano T, Watanabe K, Ohashi M, Yamazaki A, Ito T (2016). Surgical treatment for osteoporotic thoracolumbar vertebral collapse using vertebroplasty with posterior spinal fusion: a prospective multicenter study. Int Orthop.

[16] Yasuda T, Kawaguchi Y, Suzuki K, Nakano M, Seki S, Watabnabe K (2017). Five-year follow up results of posterior decompression and fixation surgery for delayed neural disorder associated with osteoporotic vertebral fracture. Medicine (Baltimore).

[17] Sudo H, Ito M, Kaneda K, Abumi K, Kotani Y, Nagahama K (2013). Anterior decompression and strut graft versus posterior decompression and pedicle screw fixation with vertebroplasty for osteoporotic thoracolumbar vertebral collapse with neurologic deficits. Spine J.

[18] Kashii M, Yamazaki R, Yamashita T, Okuda S, Fujimori T, Nagamoto Y (2013). Surgical treatment for osteoporotic vertebral collapse with neurological deficits: retrospective comparative study of three procedures--anterior surgery versus posterior spinal shorting osteotomy versus posterior spinal fusion using vertebroplasty. Eur Spine J.

[19] Watanabe K, Katsumi K, Ohashi M, Shibuya Y, Hirano T, Endo N, Kaito T, Yamashita T, Fujiwara H, Nagamoto Y, Matsuoka Y, Suzuki H, Nishimura H, Terai H, Tamai K, Tagami A, Yamada S, Adachi S, Yoshii T, Ushio S, Harimaya K, Kawaguchi K, Yokoyama N, Oishi H, Doi T, Kimura A, Inoue H, Inoue G, Miyagi M, Saito W, Nakano A, Sakai D, Nukaga T, Ikegami S, Shimizu M, Futatsugi T, Ohtori S, Furuya T, Orita S, Imagama S, Ando K, Kobayashi K, Kiyasu K, Murakami H, Yoshioka K, Seki S, Hongo M, Kakutani K, Yurube T, Aoki Y, Oshima M, Takahata M, Iwata A, Endo H, Abe T, Tsukanishi T, Nakanishi K, Watanabe K, Hikata T, Suzuki S, Isogai N, Okada E, Funao H, Ueda S, Shiono Y, Nojiri K, Hosogane N, Ishii K. Surgical outcomes of spinal fusion for osteoporotic vertebral fracture in the thoracolumbar spine: comprehensive evaluations of 5 typical surgical fusion techniques. J Orthop Sci. 2019. 10.1016/j.jos.2019.07.018. 10.1016/j.jos.2019.07.018 31445858

[20] Fujiwara A, Kobayashi N, Saiki K, Kitagawa T, Tamai K, Saotome K (2003). Association of the Japanese Orthopaedic Association score with the Oswestry disability index, Roland-Morris disability questionnaire, and short-form 36. Spine (Phila Pa 1976).

[21] McLain RF, Sparling E, Benson DR (1993). Early failure of short-segment pedicle instrumentation for thoracolumbar fractures. A preliminary report. J Bone Joint Surg Am.

[22] Lazaro BC, Deniz FE, Brasiliense LB, Reyes PM, Sawa AG, Theodore N (2011). Biomechanics of thoracic short versus long fixation after 3-column injury. J Neurosurg Spine.

[23] Peters T, Chinthakunta SR, Hussain M, Khalil S (2014). Pedicle screw configuration for thoracolumbar burst fracture treatment: short versus long posterior fixation constructs with and without anterior column augmentation. Asian Spine J.

[24] Tezeren G, Kuru I (2005). Posterior fixation of thoracolumbar burst fracture: short-segment pedicle fixation versus long-segment instrumentation. J Spinal Disord Tech.

[25] Altay M, Ozkurt B, Aktekin CN, Ozturk AM, Dogan O, Tabak AY (2007). Treatment of unstable thoracolumbar junction burst fractures with short- or long-segment posterior fixation in magerl type a fractures. Eur Spine J.

[26] Ugras AA, Akyildiz MF, Yilmaz M, Sungur I, Cetinus E (2012). Is it possible to save one lumbar segment in the treatment of thoracolumbar fractures?. Acta Orthop Belg.

[27] Oner CF, Verlaan JJ. Burst Fracture Treatment. In: Bellabarba C, Kandziora F, editors. AOSpine Masters Series, Volume 6: Thoracolumbar Spine Trauma. Stuttgart: Thieme; 2015. p. 97–108.

[28] Ohtori S, Inoue G, Orita S, Yamauchi K, Eguchi Y, Ochiai N, Kishida S, Kuniyoshi K, Aoki Y, Nakamura J, Ishikawa T, Miyagi M, Kamoda H, Suzuki M, Kubota G, Sakuma Y, Oikawa Y, Inage K, Sainoh T, Takaso M, Toyone T, Takahashi K (2013). Comparison of teriparatide and bisphosphonate treatment to reduce pedicle screw loosening after lumbar spinal fusion surgery in postmenopausal women with osteoporosis from a bone quality perspective. Spine (Phila Pa 1976).

[29] Tsuchie H, Miyakoshi N, Kasukawa Y, Nishi T, Abe H, Segawa T, Shimada Y (2016). The effect of teriparatide to alleviate pain and to prevent vertebral collapse after fresh osteoporotic vertebral fracture. J Bone Miner Metab.

[30] Wu ZX, Gong FT, Liu L, Ma ZS, Zhang Y, Zhao X, Yang M, Lei W, Sang HX (2012). A comparative study on screw loosening in osteoporotic lumbar spine fusion between expandable and conventional pedicle screws. Arch Orthop Trauma Surg.

[31] Sawakami K, Yamazaki A, Ishikawa S, Ito T, Watanabe K, Endo N (2012). Polymethylmethacrylate augmentation of pedicle screws increases the initial fixation in osteoporotic spine patients. J Spinal Disord Tech.

[32] El Saman A, Meier S, Sander A, Kelm A, Marzi I, Laurer H (2013). Reduced loosening rate and loss of correction following posterior stabilization with or without PMMA augmentation of pedicle screws in vertebral fractures in the elderly. Eur J Trauma Emerg Surg.

